# Bioinformatic Prediction of WSSV-Host Protein-Protein Interaction

**DOI:** 10.1155/2014/416543

**Published:** 2014-05-19

**Authors:** Zheng Sun, Shihao Li, Fuhua Li, Jianhai Xiang

**Affiliations:** ^1^Key Laboratory of Experimental Marine Biology, Institute of Oceanology, Chinese Academy of Sciences, 7 Nanhai Road, Qingdao 266071, China; ^2^National & Local Joint Engineering Laboratory of Ecological Mariculture, 7 Nanhai Road, Qingdao 266071, China

## Abstract

WSSV is one of the most dangerous pathogens in shrimp aquaculture. However, the molecular mechanism of how WSSV interacts with shrimp is still not very clear. In the present study, bioinformatic approaches were used to predict interactions between proteins from WSSV and shrimp. The genome data of WSSV (NC_003225.1) and the constructed transcriptome data of *F. chinensis* were used to screen potentially interacting proteins by searching in protein interaction databases, including STRING, Reactome, and DIP. Forty-four pairs of proteins were suggested to have interactions between WSSV and the shrimp. Gene ontology analysis revealed that 6 pairs of these interacting proteins were classified into “extracellular region” or “receptor complex” GO-terms. KEGG pathway analysis showed that they were involved in the “ECM-receptor interaction pathway.” In the 6 pairs of interacting proteins, an envelope protein called “collagen-like protein” (WSSV-CLP) encoded by an early virus gene “wsv001” in WSSV interacted with 6 deduced proteins from the shrimp, including three integrin alpha (ITGA), two integrin beta (ITGB), and one syndecan (SDC). Sequence analysis on WSSV-CLP, ITGA, ITGB, and SDC revealed that they possessed the sequence features for protein-protein interactions. This study might provide new insights into the interaction mechanisms between WSSV and shrimp.

## 1. Introduction

WSSV is one of the most dangerous pathogens that are destructive to penaeid shrimp, which results in up to 100% mortality in commercial shrimp farms [1]. In order to find out feasible approaches dealing with the virus, more and more studies have been carried out in crustaceans in last decade. The transcriptional profile of WSSV genes in shrimp was detected by DNA microarray and some early genes were discovered [2]. Many host genes and proteins responding to WSSV infection were also identified through large scale approaches [3–7]. From these studies, a lot of host genes and proteins were found upregulated or downregulated after WSSV infection. However, evidence on the direct interaction between WSSV and the host proteins is still urgent for understanding the pathogenesis of WSSV in shrimp.

Previous studies have noticed the importance of genes and proteins involved in WSSV/shrimp interaction. The beta-integrin, a cell surface molecule, was found to be a possible cellular receptor for WSSV infection by interacting with WSSV envelope protein VP187 [8]. Neutralization analysis with antibodies revealed that the WSSV envelope proteins VP68, VP281, and VP466 played roles in WSSV infection in shrimp [9]. The activity of the immediate-early gene* ie1* of WSSV could be upregulated by shrimp NF-*κ*B through binding to the promoter of* ie1* gene [10]. Although these data provide us some useful information about WSSV infection mechanism, it is still not very clear about the molecular mechanism of WSSV infection. At present, the whole genome of two different WSSV isolates has been sequenced, one of which is about 293 kb encoding 184 open reading frames (ORFs) [11] and another one is about 305 kb containing 181 ORFs [12]. Meanwhile, high-throughput data on the Chinese shrimp transcriptome has also been published, which contains 64,188,426 Illumina reads and isolates 46,676 unigene sequences [7]. Under this condition, bioinformatic analysis will provide a highly effective approach for identifying genes and proteins involved in WSSV/shrimp interaction based on the public protein-protein interaction (PPI) databases.

The most widely used PPI databases mainly include the Search Tool for the Retrieval of Interacting Genes/Proteins (STRING), the Database of Interacting Proteins (DIP), and Reactome. STRING is a database of known and predicted protein interactions based on the sources derived from the genomic context, high-throughput experiments, coexpression, and previous knowledge [13]. DIP is a database that records experimentally proved PPIs and provides scientific community with a comprehensive and integrated tool for browsing and efficiently extracting information about protein interactions and interaction networks in biological processes [14]. Another database, Reactome, is a manually curated and peer-reviewed pathway database [15], providing pathway related PPI information. These databases are useful resources for analyzing PPIs.

In the present study, the PPIs between WSSV and shrimp were predicted through searching the databases of STRING, DIP, and Reactome by using the data of WSSV genome sequences downloaded from the GenBank and the transcriptome data of the Chinese shrimp* Fenneropenaeus chinensis* sequenced by our lab [7]. Forty-four pairs of PPIs between WSSV and the shrimp were totally predicted. Further analysis on PPIs between the WSSV envelope proteins and the shrimp membrane proteins was carried out and the WSSV collagen like protein (WSSV-CLP) was predicted interacting with integrin and syndecan protein of the shrimp.

## 2. Materials and Methods

### 2.1. Data Preparation

WSSV genome data (accession number: NC_003225) was downloaded from the GenBank (http://www.ncbi.nlm.nih.gov/genome) and called “WSVG” in the present study. The transcriptome data of the Chinese shrimp* Fenneropenaeus chinensis* was sequenced by our lab [7] and called “FCT” in this study. Three PPI databases were localized using related data downloaded from websites. The information of downloaded files and databases was listed in Table 1.

### 2.2. Bioinformatic Analyses

#### 2.2.1. Screening of WSSV/Shrimp Interaction Proteins

Before screening, the BLAST program was downloaded for localization from the NCBI website (ftp://ftp.ncbi.nlm.nih.gov/blast/executables/blast+/LATEST/). The procedure of screening of WSSV/shrimp interaction proteins consisted of two steps. The first step was searching for similar sequences between WSVG data (or FCT data) and three PPI databases, including DIP (http://dip.doe-mbi.ucla.edu) database, Reactome (http://www.reactome.org/ReactomeGWT/entrypoint.html) database, and STRING (http://string-db.org/) database using the localized BLASTx program, respectively. The WSVG data and the FCT data were used as query sequences and the PPI databases were used as references (*E* value cutoff: <10^−5^). The output data were designated as WSVG_STRING_SBJCT, FCT_STRING_SBJCT, WSVG_DIP_SBJCT, FCT_DIP_SBJCT, WSVG_Reactome_SBJCT, and FCT_Reactome_SBJCT, respectively. The second step was to predict the potential interacting proteins using similar sequences data generated in the first step. The interacting proteins predicted by STRING were identified after comparison of the WSVG_STRING_SBJCT and FCT_STRING_SBJCT in the STRING interaction relation table. Similarly, DIP and Reactome databases were used to predict the interacting proteins following the above described procedures. In addition, interactions between predicted WSSV proteins and whole WSSV proteins and predicted shrimp proteins and all shrimp proteins were analyzed using the above-mentioned methods.

#### 2.2.2. Analysis of Predicted Interacting Proteins

Interacting proteins predicted in Section 2.2.1 were used for gene ontology (GO) analysis by Blast2GO (http://www.blast2go.org/). Subsequent pathway analysis was carried out using the online software KAAS-KEGG (http://www.genome.jp/tools/kaas/). Alignment analyses of the interested genes or proteins were performed by online software ClustalX (http://www.ebi.ac.uk/Tools/msa/clustalw2/), and functional domains and protein binding sites of interested proteins were analyzed by Conserved Domains Database (CDD) search program (http://www.ncbi.nlm.nih.gov/cdd). O-linked glycosylation sites in SDC protein were predicted using the online software NetOGlyc 3.1 (http://www.cbs.dtu.dk/services/NetOGlyc/).

## 3. Results

### 3.1. Prediction of WSSV/Shrimp Interacting Proteins

Forty-four pairs of WSSV/shrimp interacting proteins, including 38 pairs STRING predicted PPIs, 32 pairs DIP predicted PPIs, and 39 pairs Reactome predicted PPIs, were totally predicted after screening of the three PPI databases using WSVG and FCT data (Table 2). Seven deduced proteins encoded by WSSV genes, including wsv001 (collagen like protein), wsv026 (hypothetical protein), wsv067 (thymidylate synthetase), wsv112 (dUTP diphosphatase), wsv128 (hypothetical protein), wsv172 (Ribonucleoside-diphosphate reductase large chain), and wsv188 (ribonucleoside-diphosphate reductase small chain), probably interacted with 32 proteins encoded by the shrimp genes.

Possible interactions between predicted proteins and their endogenous proteins were also predicted. For predicted WSSV proteins, five out of seven predicted members showed interactions with other WSSV proteins. For predicted shrimp proteins, 31 out of 32 members showed interactions with other shrimp proteins (see Table S1 in Supplementary Material available online at http://dx.doi.org/10.1155/2014/416543).

### 3.2. Potential Interaction between WSSV Envelope Proteins and Shrimp Membrane Proteins

**Figure 1:**
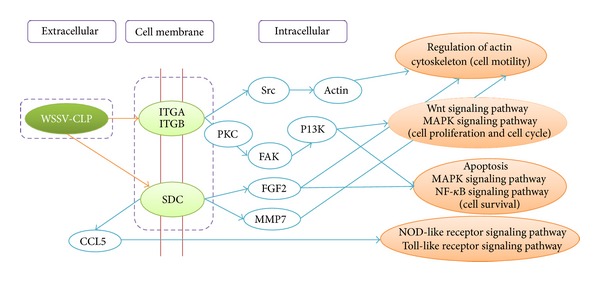
A schematic diagram was drawn to describe the predicted protein-protein interactions between WSSV-CLP and membrane proteins from the shrimp and the intracellular signaling pathways induced by the interactions. The diagram was drawn based on the KEGG pathway map04510 (focal adhesion) and map04512 (ECM-receptor interaction). WSSV-CLP: WSSV collagen like protein; ITGA: integrin alpha; ITGB: integrin beta; SDC: syndecan; CCL5: chemokine ligand 5; PKC: protein kinase C; FAK: focal adhesion kinase; PI3K: phosphoinositide 3-kinase; FGF2: fibroblast growth factor 2; MMP7: matrix metalloproteinase-7.

GO analysis (based on cellular components, level 2) showed that 5 WSSV proteins and 23 shrimp proteins had GO annotation (Table S2). Among them, a WSSV envelope protein “collagen-like protein” (WSSV-CLP, NP_477523.1) encoded by wsv001 belonged to the GO-term of “extracellular region.” Six proteins from the shrimp, including three integrin alpha proteins (ITGAs) (s_12679, s_18988, and s_3390), two integrin beta proteins (ITGBs) (s_1537 and s_16763), and one syndecan protein (SDC) (s_2496) showed interactions with WSSV-CLP (Table 2). The six proteins were classified into the “extracellular region” or “receptor complex” GO-terms (Table S2). The subsequent KEGG analysis (Table S2) revealed that the interactions between WSSV-CLP and ITGA/ITGB/SDC were involved in the “ECM-receptor interaction” and “focal adhesion” pathways (Figure 1).

Further analysis on endogenous proteins interacting with WSSV-CLP or ITGA/ITGB/SDC was carried out. As shown in Table 3, WSSV-CLP could interact with two other WSSV proteins, including ribonucleotide reductase small subunit (wsv188) and ribonucleotide reductase large subunit RR1 (wsv172). In shrimp, 20 endogenous proteins were found interacting with ITGA, ITGB, or SDC. The endogenous proteins interacting with ITGA mainly included fms-related tyrosine kinase 4, fibronectin 1, integrin beta 1, talin 1, insulin-like receptor, integrin alpha 1, myospheroid, ultraspiracle, and ecdysone receptor (Table 3). Endogenous proteins interacting with ITGB contained fibronectin 1, integrin alpha 1, integrin alpha 4, integrin alpha 5, integrin-linked kinase, lysosomal-associated membrane protein 1, and Lysosomal-associated membrane protein 2 (Table 3). Endogenous proteins interacting with SDC were annotated as calcium/calmodulin-dependent protein kinase, dally-like, kon-tiki, and* Drosophila* transcripts CG3194 and CG9298 (Table 3).

### 3.3. Analysis on the Amino Acid Sequence of WSSV-CLP

The WSSV-CLP, encoding by the published WSSV collagen like protein gene, was predicted possessing three collagen triple helix repeat domains. In the amino acid sequence of WSSV-CLP, the Gly-X-X (GXX, where X represents any amino acid) motifs were widely distributed from 161 aa to 1327 aa, where the GXXGER motif appeared for 21 times and the GXXGEN motif appeared for eight times (data not shown).

### 3.4. Sequence Analyses on ITGAs and ITGBs

**Figure 2:**
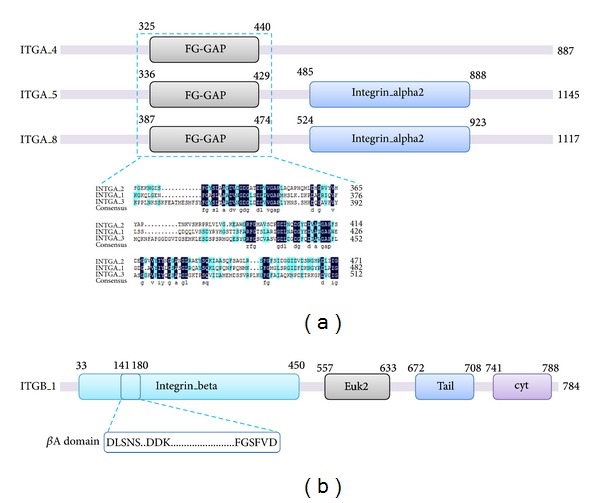
Schematic description of domains in ITGA (a) and ITGB (b). The FG-GAP domain, integrin alpha 2 domain, integrin beta domain, *β*A domain, EuK2 domain, tail (integrin beta tail) domain, and cyt (integrin beta cytoplasmic) domain located in ITGA and ITGB were shown with their beginning residue sites and stopping residue sites. The detailed sequence information of FG-GAP domain in ITGA_4, ITGA_5, and ITGA_8 were listed and aligned. The common feature of *β*A domain in ITGB_1 integrin beta domain was shown in a green box.

Three ITGA homologs and two ITGB homologs were identified as WSSV-CLP interaction proteins. They were designated here as ITGA_4 (accession number: KC715736), ITGA_5 (accession number: KC715737), ITGA_8 (accession number: KC715738), ITGB_1 (accession number: KC715739), and ITGB_6 (accession number: KC715740) according to the BlastX annotation, respectively. Sequence analysis revealed that ITGA_5, ITGA_8, and ITGB_1 contained complete open reading frame (ORF), while ITGA_4 and ITGB_6 had partial ORF (see online submitted sequences). Alignment of above sequences by ClustalX showed that they shared poor similarity (data not shown). CDD analysis revealed that all the three ITGAs contained FG-GAP repeats in the N terminus (Figure 2(a)). In addition, ITGA_5 and ITGA_8 also possessed the integrin alpha 2 domain. ITGB_1 contained four conserved domains, including an integrin beta domain, an integrin beta tail domain (tail, pfam07965), an integrin beta cytoplasmic domain (cyt, pfam08725), and a conserved domain of eukaryotic metallothioneins family (Euk2, pfam12809) Metallothi_Euk2 (Figure 2(b)). In the Integrin beta domain, there is a *β*A domain (amino acids number 141–180), which is a member of the type A domain superfamily containing a prototype molecule called von Willebrand factor (vWF). The *β*A domain contained the conserved ligand-binding sites of DLSNS, DDK, and FGSFVD (Figure 2(b)).

### 3.5. Sequence Analysis on SDC

**Figure 3:**
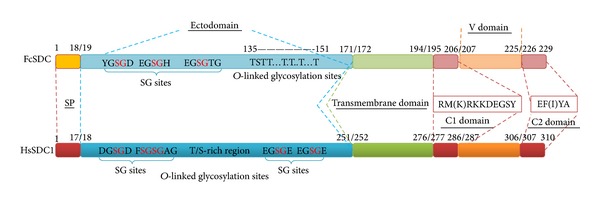
A compared schematic structure of syndecan from shrimp (FcSDC) and syndecan-1 (accession number: NP_001006947) from* Homo sapiens *(HsSDC1). The signal peptide (SP), syndecan conserved domains (transmembrane domain, C1 domain and C2 domain), and syndecan-type specific domains (ectodomain and V domain) were displayed with the number of their boundary amino acid residues. The potent glycosylation sites, including SG sites and predicted* O*-linked glycosylation sites, were shown. The sequence information of C1 domain and C2 domain was also listed.

The SDC (accession number: KC733458) isolated from the FCT data contained a complete ORF and the deduced amino acid sequence had a signal peptide and five conserved domains, including ectodomain, transmembrane domain, C1 domain, C2 domain, and V domain (Figure 3), which showed the typical characteristics of syndecans. The C1 domain showed a conserved sequence feature of RMK(R)KKDEGSY, and the C2 domain had a conserved sequence feature of EF(I)YA. It showed high similarities in transmembrane domain, C1 domain and C2 domain among shrimp syndecan (FcSDC), and syndecan 1 from mammals (HsSDC1). However, the ectodomain and V domain showed low conservation among them. One serine residue and seven threonine residues were predicted in the C terminus of the ectodomain with the potential to be* O*-linked glycosylated. Three Ser-Gly (SG) sites, including YGSGD, EGSGH, and EGSGT, located in the N terminus of the ectodomain of FcSDC. In the ectodomain of HsSDC1, potential* O*-linked glycosylation sites mainly located in the T/S-rich region and four SG sites also existed (Figure 3).

## 4. Discussions

Exploitation of protein-protein interaction information with bioinformatic approaches provides an effective way to analyze high-throughput experimental data and has been widely applied in distinct organisms [16, 17]. With the genome data of WSSV [11, 12] and abundant transcriptome data from shrimp, it becomes possible to identify WSSV/shrimp interacting proteins with developed bioinformatic techniques. In the present study, we screened all possible WSSV/shrimp interacting proteins and a total of seven putative proteins encoded by WSSV were predicted interacting with deduced proteins from shrimp. Although only the WSSV-CLP was focused for further analysis, the other six putative proteins from WSSV also provided important information. Four of them were annotated as thymidylate synthetase, dUTP diphosphatase, ribonucleoside-diphosphate reductase large chain, and ribonucleoside-diphosphate reductase small chain, respectively. Thymidylate synthase [18] and dUTP diphosphatase [19] could generate direct or indirect substrates for DNA synthesis and reduce the risk of DNA repair. Ribonucleoside-diphosphate reductase is also an important enzyme for DNA replication [20–22]. As WSSV original proteins, these enzymes might play the key roles during WSSV genome replication. Identification of host proteins interacting with these enzymes provided new clues to develop disease control techniques.

The invasion route that WSSV infects the host cells is the point that we are interested in. After GO classification and pathway analysis, the previously reported WSSV envelope protein WSSV-CLP was predicted interacting with ITGA, ITGB, and SDC from the shrimp. Endogenous proteins which might interact with WSSV-CLP, ITGA, ITGB, and SDC were also screened, which provided new insights into how WSSV interact with host cells. In the present study, WSSV-CLP and its interacting proteins in shrimp were further analyzed. The WSSV-CLP was first reported after whole genome sequencing [12] and then described as an early viral gene encoding an envelope protein [23]. However, there is still no report revealing WSSV-CLP interaction proteins from the hosts. In mammalian, monoclonal antibodies of *α*2*β*1 integrin could block the collagen-induced morphogenesis of human mammary epithelial cells [24]. The influence might be caused by interaction between *α*2*β*1 integrin and specific sites of collagen because the *α*2*β*1 integrin was a cell-surface receptor for collagen [25] and the synthetic collagen-mimetic peptides, GXXGER and GXXGEN, showed binding affinities with *α*2*β*1 integrin [26, 27]. The collagen binding site of *α*2*β*1 integrin was localized to the *α*2 von Willebrand factor type A (vWFA) domain [28]. The reported *β*-integrin in shrimp could also bind WSSV and the recombinant extracellular region of the *β*-integrin could partially block WSSV infection to shrimp [29]. This binding was deemed as an interaction of *β*-integrin with RGD motif presented in WSSV proteins [29] and previous study revealed that an RGD motif containing protein in WSSV, VP187 encoded by wsv209, was a potent ligand of *β*-integrin in shrimp [8]. In the present study, the WSSV-CLP was predicted interacting with ITGA and ITGB from the shrimp. Sequence analysis revealed that WSSV-CLP contained 21 GXXGER motifs and 8 GXXGEN motifs in the collagen triple helix repeat domain. These motifs might be the binding sites of WSSV-CLP with integrin according to the previous studies in mammalians. The FG-GAP repeats found in the N terminus of ITGA chains have been shown to be important for ligands binding [30], such as collagens, fibronectins, fibrinogen, and laminins [31]. A *β*A domain existed in the integrin beta domain of ITGB_1. It was a RGD binding site that contained the conserved ligand-binding sites of DLSNS, DDK, and FGSFVD and could be recognized by VP187 protein of WSSV [8]. These data might indicate that ITGA_5 and ITGB_1 are possible receptors of WSSV in shrimp. The function of ITGAs and ITGBs in WSSV infection route is worthy to be investigated further.

Syndecan was another predicted protein in shrimp which could interact with WSSV-CLP. Synergistic interaction between syndecan and integrin in cell adhesion [32] and cell spreading [33] was already reported. Furthermore, syndecan-1 in mammals could support the integrin *α*2*β*1-mediated adhesion to collagen [34]. The SDC isolated from shrimp displayed a similar sequence feature with syndecan-1 from* Homo sapiens*. The SG sites (YGSGD, EGSGH, and EGSGT) presented in the N terminus of SDC ectodomain shared great sequence and location similarities with the SG sites in syndecan-1 from* Homo sapiens*, which possessed the SG sites such as FSGSGTG, DGSGD, EGSGE, and ETSGE responsible for HS or CS chains formation [35]. In addition, the predicted* O*-linked glycosylation sites located in the C terminus of SDC ectodomain also provided possible glycosylation sites to generate HS or CS chains. These characteristics were probably responsible for WSSV-CLP binding during infection. In the present study, both integrin and SDC were predicted having interaction with WSSV-CLP. Integrin and SDC are regarded as membrane receptors. Interaction of WSSV-CLP with integrin or SDC might lead to the regulation of actin for cell motility or initiate the intracellular signaling pathways, such as MAPK, NF-kappa B signaling pathway, which are responsive to WSSV challenge in shrimp [35–38].

In conclusion, the present study identified the WSSV/shrimp interacting proteins by bioinformatic analysis on the high-throughput gene data. The predicted interactions between WSSV-CLP and integrins and between WSSV-CLP and SDC, which might be either independent interaction or synergistic interaction, provided possible invasion approaches during WSSV infection to host cells. Moreover, these interactions could also lead to intracellular signaling pathways initiated by integrins or SDC as described in Figure 1. Further experimental confirmation is necessary for the prediction results in the future.

## Supplementary Material

Table S1 showed endogenous proteins interacting with predicted proteins from WSSV and shrimp. Five out of seven predicted WSSV proteins exhibit interactions with other WSSV proteins, while 31 out of 32 predicted shrimp proteins show interactions with other shrimp proteins.
Table S2 showed GO and pathway analysis of interacting proteins between WSSV and the Chinese shrimp. GO analysis indicated that five predicted WSSV proteins and 23 predicted shrimp proteins had GO annotation. The following KEGG analysis revealed pathways that predicted proteins were involved in.

## Figures and Tables

**Table 1:** The downloaded information used for database construction in the present study.

Database	Download address	Definition	Data type	Name used in the present study
Reactome	http://www.uniprot.org/downloads	UniProtKB/TrEMBL	Reference sequence (Fasta)	Reactome gene database
	http://www.reactome.org/download/all_interactions.html	Drosophila melanogaster protein-protein interaction pairs	Interaction relation table	Reactome interaction relation table

DIP	http://dip.doe-mbi.ucla.edu/dip/Download.cgi?SM=4	fasta20120218.seq	Reference sequence (Fasta)	DIP gene database
	http://dip.doe-mbi.ucla.edu/dip/Download.cgi?SM=3	dip20120818.txt	Interaction relation table	DIP interaction relation table

STRING9.0	http://string-db.org/newstring_cgi/show_download_ page.pl?UserId=DNt0ic8Ndem8&sessionId=heGmVXVt8w_a	protein.sequences.v9.0.fa.gz	Reference sequence (Fasta)	STRING gene database
		protein.actions.v9.0.txt.gz	Interaction relation table	STRING interaction relation table

**Table 2:** Predicted interacting proteins between WSSV and the Chinese shrimp.

WSSV_ID	Annotation	Interacting protein in FCT		Database		
		FCT_ID	Annotation	STRING	DIP	Reactome
wsv001	Collagen like protein	s_18988	Integrin alpha 4	/	/	*√*
		s_12679	Integrin alpha 5			
		s_3390	Integrin alpha 8			
		s_1537	Integrin beta 1			
		s_16763	Integrin beta 6			
		s_2496	Syndecan			

wsv026	hypothetical protein	s_5219	Putative methylase/helicase	*√*	/	*√*

wsv067	thymidylate synthetase	s_3110	N/A	*√*	*√*	*√*
		s_13286	N/A			
		s_2426	Glycine dehydrogenase			
		s_5707	Inosine triphosphatase			
		s_2204	Proliferating cell nuclear antigen			
		s_3879	Ribonucleotide reductase 1			
		s_1099	Thymidine kinase 1			

wsv112	dUTP diphosphatase	s_16017	Nucleoside diphosphate kinase	*√*	*√*	*√*
		s_20130	Thymidylate kinase			
		s_5707	Inosine triphosphatase			
		s_60	Nucleoside diphosphate kinase			
		s_3879	Ribonucleotide reductase 1			
		s_1099	Thymidine kinase 1			

wsv128	hypothetical protein	s_15702	Beta-transducin repeat containing protein	*√*	/	/
		s_3485	Hypothetical protein			
		s_5154	Hypothetical protein			
		s_6901	Hypothetical protein			
		s_13825	Hypothetical protein			

wsv172	Ribonucleoside-diphosphate reductase large chain	s_4560	Adenylate kinase	*√*	*√*	*√*
		s_6839	Minichromosome maintenance complex component 4			
		s_60	Nucleoside diphosphate kinase			
		s_18721	Cell division control protein 45 homolog			
		s_18944	Probable thymidylate synthase			
		s_4876	Guanylate kinase			
		s_2904	Probable uridylate kinase			
		s_1744	Ribonucleoside-diphosphate reductase small chain			
		s_20130	Thymidylate kinase			

wsv188	Ribonucleoside-diphosphate reductase small chain	s_5545	Adenylate kinase 3	*√*	*√*	*√*
		s_20130	Thymidylate kinase			
		s_16017	Nucleoside diphosphate kinase			
		s_2904	Probable uridylate kinase			
		s_386	Adenylate kinase isoenzyme			
		s_4876	Guanylate kinase			
		s_20711	Conserved hypothetical protein (*Aedes aegypti*)			
		s_60	Nucleoside diphosphate kinase			
		s_4560	Adenylate kinase			
		s_3879	Ribonucleotide reductase 1			

*Note*. “/” means absence of prediction in relevant database, while “*√*” means presence of prediction in relevant database.

**Table 3:** Endogenous proteins interacting with WSSV-CLP, ITGA, ITGB, and SDC.

Predicted gene ID	Predicted gene annotation	Gene ID of endogenous interacting proteins	Annotation of endogenous interacting proteins
s_12679	Integrin alpha 5	s_17653	fms-related tyrosine kinase 4
		s_25665	Fibronectin 1
		s_1537	Integrin beta 1
		s_1294	Talin 1

s_18988	Integrin alpha 4	s_21444	Insulin-like receptor
		s_1537	Integrin beta 1
		s_5849	Integrin alpha 1
		s_2170	Myospheroid
		s_22423	Ultraspiracle

s_3390	Integrin alpha 8	s_30884	Ecdysone receptor
		s_21444	Insulin-like receptor
		s_1537	Integrin beta 1
		s_5849	Integrin alpha 1
		s_2170	Myospheroid

s_16763	Integrin beta 6	s_23243	Fibronectin 1
		s_5849	Integrin alpha 1
		s_6902	Integrin alpha 4
		s_26063	Integrin alpha 5

s_1537	Integrin beta 1	s_8242	Integrin-linked kinase
		s_5849	Integrin alpha 1
		s_7013	Lysosomal-associated membrane protein 1
		s_11802	Lysosomal-associated membrane protein 2
		s_26063	Integrin alpha 5

s_2496	Syndecan	s_24189	CG3194
		s_3573	CG9298
		s_18063	Calcium/calmodulin-dependent protein kinase
		s_18648	Dally-like
		s_7954	Kon-tiki

wsv001	Collagen triple helix repeat protein	wsv188	Ribonucleotide reductase small subunit
		wsv172	Ribonucleotide reductase large subunit RR1
